# Spontaneous Hydropneumothorax After Pleurodesis in a Patient With Pulmonary Lymphangioleiomyomatosis With Associated Endometrial Cancer

**DOI:** 10.7759/cureus.64723

**Published:** 2024-07-17

**Authors:** Sabastain F Forsah, Gauvain Kankeu Tonpouwo, Derek Ugwendum, Marvel Changoh, Divine Besong Arrey Agbor, Bibi S Razak, Justin Muego, Keith Diaz

**Affiliations:** 1 Internal Medicine, Richmond University Medical Center, Staten Island, USA; 2 Pulmonary and Critical Care, Richmond University Medical Center, Staten Island, USA

**Keywords:** mtor activation, tuberous sclerosis, endometrial cancer, lymphangioleiomyoma, angiomyolipomas, hydropneumothorax, lymphangioleiomyomatosis

## Abstract

Lymphangioleiomyomatosis (LAM) is a rare cystic disease that occurs due to the abnormal proliferation of smooth muscle-like cells. It primarily affects the lungs but can also have extrapulmonary manifestations such as lymphangioleiomyoma and angiomyolipomas. It is more common in young women of childbearing age, with female sex hormones contributing to the disease course. LAM can develop either through sporadic mutations or through genetic inheritance of the tuberous sclerosis complex (TSC) genes. TSC, LAM, and endometrial cancer are associated with mTOR pathway activation, which can explain why these diseases can co-exist, although the co-existence of LAM and endometrial cancer in the same patient is very rare. Due to the cystic nature of LAM, pneumothorax most often occurs at least once during the course of the disease, and most times, it is the first manifestation observed in LAM. These patients are also at high risk for recurrent pneumothorax, and when that occurs, pleurodesis is indicated. Unfortunately, pleurodesis still does not preclude a pneumothorax from occurring. We present the case of a female patient with LAM and endometrial cancer who was found to have an incidental spontaneous hydropneumothorax after pleurodesis. Patients with LAM should be closely monitored for the possible development of other mTOR-associated diseases. Moreover, when performing pleurodesis for recurrent pneumothorax in very high-risk patients, the procedure with the lowest recurrence rate should be utilized.

## Introduction

Lymphangioleiomyomatosis (LAM) is a rare, multisystem, progressive, cystic disease, with the lungs being the most commonly affected organ. The cystic lesions in LAM replace the normal functional airspace, thereby causing the destruction of the lung tissues. This can cause a progressive decline in lung function and, if severe enough, may necessitate a lung transplant [1]. LAM is more common in women of childbearing age [2]. Due to the cystic nature of the disease, most patients with LAM will experience at least one episode of spontaneous pneumothorax, which might be asymptomatic and found incidentally [2]. Pleurodesis is indicated for recurrent pneumothorax, and depending on the type of pleurodesis and the patient’s risk factors, pneumothorax can recur [3]. Symptomatic pulmonary LAM presents with progressive dyspnea, chylothorax, and hemoptysis. There are also extrapulmonary manifestations of LAM such as renal angiomyolipomas [4]. LAM in the female genital tract primarily affects the uterus and mimics malignant disease. It can also co-exist with an underlying malignancy of the female genital tract, although the co-existence is very rare [5,6]. Whether the lesions in the uterus are a result of metastasis or the source of cancer has been the subject of debate.

Our case is that of a female patient with uterine cancer, LAM, and pleurodesis after recurrent pneumothorax, who presented with abdominal pain for which CT of the abdomen showed an incidental hydropneumothorax. To our knowledge, hydropneumothorax occurring after pleurodesis on a background of pulmonary LAM with associated uterine malignancy in a single patient is extremely rare. The association of LAM and uterine malignancy is probably related to mTOR abnormalities.

## Case presentation

**Figure 1:**
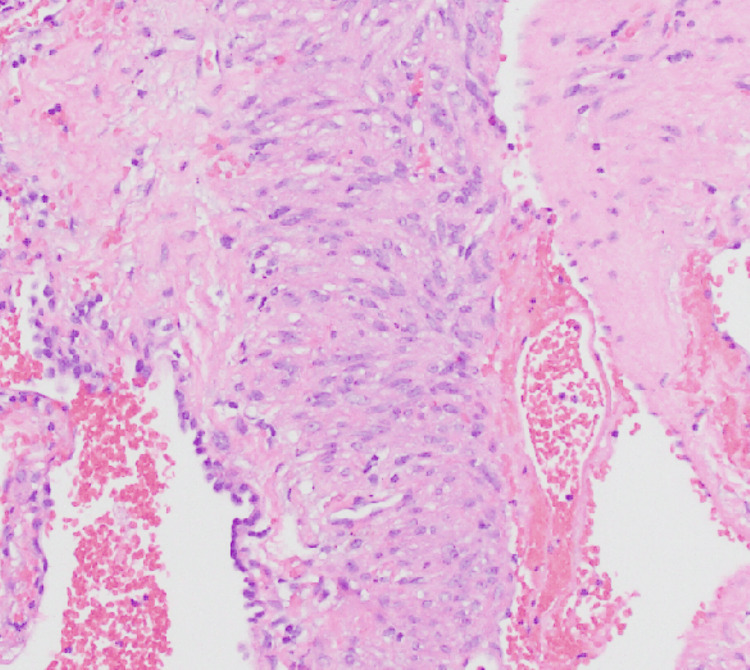
Lung pathology report revealing diffuse proliferation of abnormal smooth muscle-like cells along the alveoli, bronchioles, and lymphatic vessels with strong and diffuse positivity for smooth muscle actin. Additionally, these cells stained positive for hydroxymethylbutyrate-45. Estrogen and progesterone receptors were positive.

**Figure 2:**
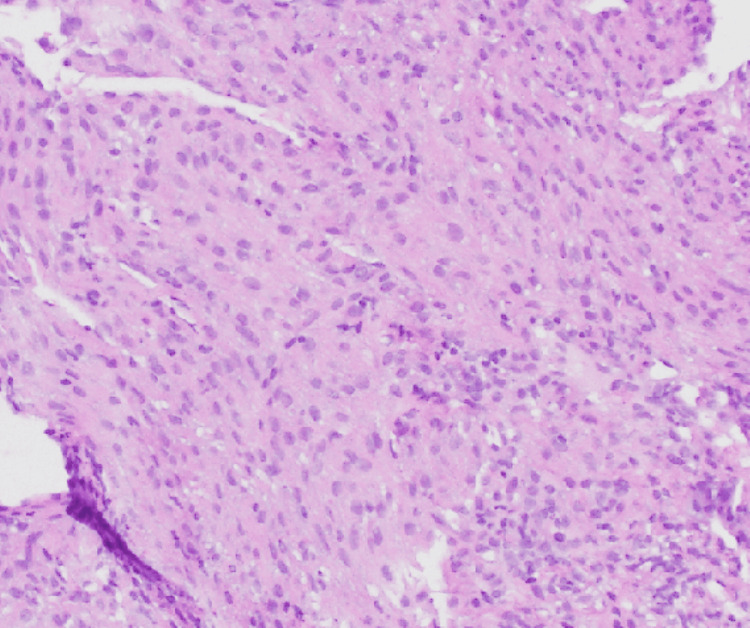
Biopsy of the retroperitoneal lymph node showing similar appearing spindle cells which also tested positive for smooth muscle actin and represent intranodal lymphangioleiomyomatosis.

A 47-year-old female presented to the emergency department with a two-day history of severe, crampy abdominal pain radiating throughout the entire abdomen and associated with diarrhea. She had no cough, chest pain, hemoptysis, or dyspnea. Her past medical history was significant for advanced endometroid endometrial cancer (on radiotherapy and recently started on pembrolizumab and lenvatinib after she progressed on carboplatin, paclitaxel, and bevacizumab) and LAM status post-bilateral video-assisted thoracoscopic surgery (VATS) with resection of bullous lung disease in the right upper lobe and bilateral mechanical pleurodesis. Pathological examination of the resected lung (Figure 1) and retroperitoneal lymph node biopsy (Figure 2), done for the staging of uterine cancer, showed LAM. Both biopsied sites were positive for estrogen and progesterone receptors. She had no smoking history.

**Figure 3:**
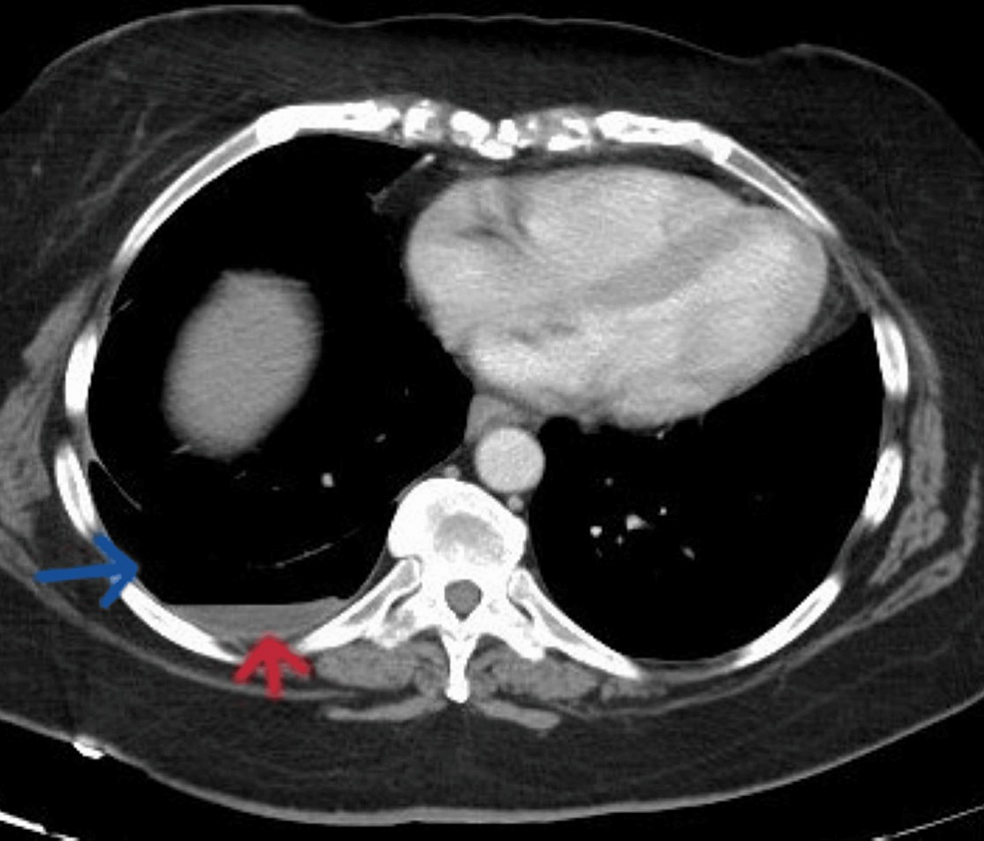
CT of the abdomen showing incidental right-sided hydropneumothorax (the red arrow shows hydrothorax and the blue arrow shows pneumothorax).

The initial vital signs were as follows: blood pressure was 115/79 mmHg, pulse rate was 105 beats/minute, temperature was 98.4°F, respiratory rate was 18 breaths/minute, and oxygen saturation was 96% on room air. Physical examination revealed decreased breath sounds in the inferior third of the right lung field with diffuse abdominal tenderness. CT of the abdomen and pelvis revealed a right-sided hydropneumothorax (Figure 3) and colitis.

**Figure 4:**
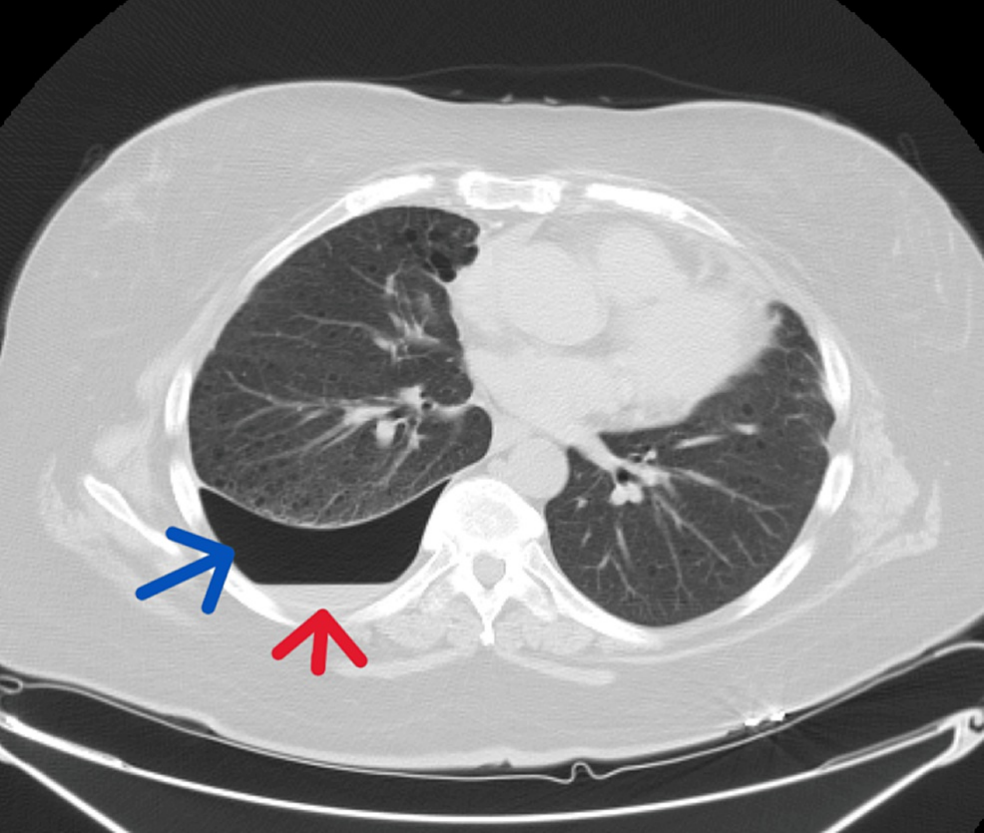
CT of the chest showing right-sided hydropneumothorax (the red arrow shows hydrothorax and the blue arrow shows pneumothorax). It also shows cystic lungs consistent with lymphangioleiomyomatosis.

A follow-up CT of the chest showed multiple, scattered, bilateral, thin-walled cysts with moderate right-sided hydropneumothorax. There was no focal consolidation, mediastinal shift, or signs of other pathology (Figure 4).

The patient refused any intervention for the hydropneumothorax, so her active issues were treated and she was discharged with bronchodilators. During the follow-up cardiothoracic surgery appointment, she was still asymptomatic, and a repeat CT of the chest showed a slight improvement in the hydropneumothorax. She also followed up with the pulmonologist, and the pulmonary function test (PFT) showed a mild-to-moderate restrictive pattern and a mild-to-moderate decreased diffusion gradient. The patient will undergo regular follow-up and imaging. She will also repeat PFTs, and if there is any evidence of declining lung function, she will be started on sirolimus.

## Discussion

The prevalence of LAM varies both regionally and nationally, and as with other rare diseases, obtaining reliable epidemiological data is difficult. The incidence of LAM ranged from 0.23 to 0.31/million women/per year, from 2004 to 2008, in the United States, the United Kingdom, and Switzerland [7]. LAM is microscopically characterized by abnormal proliferation of abnormal smooth muscle-like cells which lead to lymphatic abnormalities (lymphangioleiomyoma), renal tumors (angiomyolipomas), and progressive destruction of the small airways, causing air trapping and formation of thin-walled cysts in the lungs [8,9].

LAM occurs either sporadically or in association with tuberous sclerosis complex (TSC). LAM is less severe in TSC-LAM than sporadic LAM [4]. Sporadic LAM occurs as a result of an acquired mutation in the *TSC2* gene, and it affects 1 in 400,000 adult females with no evidence of genetic disease. Genetically, LAM occurs in 30%-40% of adult females with TSC (TSC1 or TSC2) disease [1,4]. Contrary to the sporadic form, the genetic form can also occur in males and children [1]. TSC is an autosomal dominant neurocutaneous syndrome characterized by hamartoma formation in multiple organ systems, cerebral calcifications, seizures, and cognitive defects [9].

An inactivating mutation of the tumor suppressor genes, *TSC1* or *TSC2*, leads to the mammalian target of rapamycin complex 1 (mTORC1) activation causing increased cell growth, making rapamycin and its analogs (like sirolimus) valuable treatment options for LAM [8]. The mTOR pathway also plays a role in the development of endometrial cancer with a high frequency of mutations in *PTEN* and/or *PIK3CA* [10]. Therefore, LAM, TSC, and endometrial cancer appear to share an association with mTOR pathway activation, explaining why they can occur in the same patient. Although we have not performed genetic testing for germline mutations in the mTOR pathway in our patient, the presence of both diseases is highly suggestive of activation of mTOR signaling.

Studies show that estrogen contributes to the development, growth, and spread of LAM cells. In fact, LAM progresses more rapidly during pregnancy and with the use of exogenous estrogens, but the progression slows after menopause [2]. Data suggests that LAM cells populate the lungs in a metastatic fashion, but the origin of the LAM cells is not known. However, the current hypothesis is that LAM cells might originate from the uterus. The cells then invade the lymphatic vessels and cell clusters spread to selected distant sites, especially to the lung and kidney [11,12]. Our patient had both pulmonary LAM and retroperitoneal lymph nodal LAM.

Patients with LAM can present with shortness of breath, cough, pneumothorax, hemoptysis, chyloptysis, and chylous pleural effusions [13]. Patients with pneumothorax can be asymptomatic like our patient or may present with sudden-onset breathlessness and pleuritic chest pain. Over 50% of patients with LAM have a history of pneumothorax in their clinical course, and it is often the first manifestation with a high rate of recurrence [9]. Extrapulmonary manifestations of LAM include large cystic lymphatic masses called lymphangioleiomyomas, renal angiomyolipoma, chylous ascites, and abdominal lymphadenopathy [2]. Lymphangioleiomyomas are usually localized in the mediastinum, retroperitoneum, and pelvis along the axial lymphatics. Angiomyolipomas are asymptomatic in most cases; however, multiple and large masses are more likely to cause hemorrhage and symptoms such as hematuria and flank pain [13].

The chest radiograph in patients with LAM may show pleural effusions or pneumothorax, and in advanced disease, it may show a reticulonodular pattern and cysts or bullae [9]. A high-resolution CT scan of the lung is the imaging modality of choice for LAM. It shows the presence of multiple (more than 10), well-circumscribed, round, and thin-walled cysts that are scattered in a bilateral roughly symmetric pattern, without any lobar predominance and with no other significant pulmonary involvement except for a possible hamartomatous process in patients who also have TSC [9]. Patients presenting with LAM should also undergo a thorough workup for tuberous sclerosis, gynecological malignancies, and abdominal lesions [4-6]. A lung biopsy may be needed to confirm the diagnosis of LAM in some patients. However, the diagnosis of LAM can be confirmed based on clinical, laboratory, and CT findings without a biopsy, if extrapulmonary manifestations such as angiomyolipoma, thoracic or abdominal chylous effusion, lymphangioleiomyoma, or biopsy-proven lymph node involved by LAM are present [14]. Our patient had no TSC findings and was diagnosed using imaging and biopsy. The serum level of vascular endothelial growth factor (VEGF)-D may further contribute to the noninvasive diagnosis of LAM [12]. Before considering diagnostic lung biopsy in patients with positive CT scan findings of LAM, but who have no other confirmatory clinical or extrapulmonary radiologic features, VEGF-D measurement can especially be vital to establish the diagnosis in a noninvasive manner [15].

Management includes controlling the symptoms, improving quality of life, slowing disease progression, managing complications, and lung transplantation for severe and refractory disease [14]. In addition to medical treatment, supportive care includes smoking cessation, correction of hypoxia with adequate oxygen supplementation, and prophylactic vaccination [14]. A trial of bronchodilators should be offered because about 20% of patients have a positive response to bronchodilators [9].

Sirolimus, a first-generation inhibitor of mTOR, is the only FDA-approved medication for the treatment of LAM; it improves lung function, quality of life, and functional performance [15]. According to the American Thoracic Society/Japanese Respiratory Society Clinical Practice Guidelines on Lymphangioleiomyomatosis in 2016, patients with LAM with abnormal/declining lung function should be treated with sirolimus. In patients with LAM and refractory chylous effusions, sirolimus should be used before invasive management [15]. Although starting patients like ours on sirolimus when they are already on immunosuppressive medications including immunotherapy (pembrolizumab) has the risk of further compromising their immune system, studies show that sirolimus improves programmed cell death 1 blockade efficacy and improves the immune-related gastrointestinal adverse effects such as colitis, which our patient had [16,17].

The guideline does not recommend the use of doxycycline and hormonal therapy for the treatment of LAM [15). Given the role of estrogens in the pathogenesis of LAM, there is a suggestion that hormonal therapy can be beneficial. However, there have been no clinical trials assessing the efficacy of these medications in LAM [14]. Therefore, there is room for more research on potential treatment options, including Rho inhibitors, tyrosine kinase inhibitors, matrix metalloproteinase inhibitors (doxycycline), and hormonal therapy.

LAM is commonly monitored by performing PFTs. Patients with severe lung damage who do not respond to treatment may be eligible for a lung transplant [12]. Our asymptomatic patient had a baseline PFT performed which showed a mild-to-moderate restrictive pattern with a mildly to moderately decreased diffusion gradient. She will be monitored with serial PFTs, and if there is evidence of declining lung function, she will be started on sirolimus. Sirolimus will not only treat her LAM but will also have a synergistic effect with pembrolizumab on her endometrial cancer by potentiating the therapeutic effects of pembrolizumab. At the same time, it will reduce its gastrointestinal side effects which will prevent the discontinuation of this important medication [16].

Conservative treatment could be the initial approach to the management of pneumothorax. Massive pneumothorax may require tube thoracostomy or VATS [18]. Indications for pleurodesis, a procedure performed to obliterate the pleural space, include malignant pleural effusion, recurrent pneumothorax, persistent air leak, and recurrent pleural effusions [18]. Risk factors for postoperative recurrence of spontaneous pneumothorax include age <40 years, active smoking, comorbidities (chronic obstructive pulmonary disease, emphysema, LAM), prolonged postoperative air leakage, and missed or incomplete bullectomy [4]. VATS bullectomy and pleurodesis is a reliable option for treatment of recurrent pneumothorax but it has a recurrence rate of 0%-11% [3]. Another study showed a 13% overall population recurrence rate for primary spontaneous pneumothorax treated with surgery. According to the specific surgical approaches, the recurrence rate for bullectomy plus mechanical pleurodesis was 13%, bullectomy plus pleurectomy was 6%, while bullectomy plus pleurectomy and mechanical pleurodesis was 0% [19]. Our patient had VATS with bullectomy and mechanical pleurodesis. High-risk patients like ours should, therefore, undergo the procedure with the lowest possible recurrence rate.

## Conclusions

LAM is a rare disease but can progress to cause severe lung damage requiring a lung transplant. A recurrent pneumothorax, which occurs at a high rate in LAM patients, is treated with pleurodesis, and depending on the type of pleurodesis and the patient’s risk factors, pneumothorax can recur. In patients at a high risk of recurrence, like those with LAM, the surgical approach employed should be that with the lowest recurrence rate. Due to the association of LAM and endometrial cancer with the mTOR activation pathway, patients with LAM should be monitored closely and any suspicious gynecological lesion should be investigated further to rule out malignancy. Studies should be conducted to investigate the effectiveness of sirolimus in the treatment of uterine malignancies to treat coexisting conditions with a common drug. Studies are also needed to find more therapeutic options for the management of LAM.
